# Evidence for the Worldwide Distribution of a Bile Salt Hydrolase Gene in *Enterococcus faecium* Through Horizontal Gene Transfer

**DOI:** 10.3390/ijms26020612

**Published:** 2025-01-13

**Authors:** Hiroyuki Kusada, Hideyuki Tamaki

**Affiliations:** Bioproduction Research Institute, National Institute of Advanced Industrial Science and Technology, Tsukuba 305-8566, Ibaraki, Japan

**Keywords:** bile salt hydrolase, *Enterococcus faecium*, horizontal gene transfer, probiotics, plasmid

## Abstract

Bile salt hydrolase (BSH), a probiotic-related enzyme with cholesterol-assimilating and anti-hypercholesterolemic abilities, has been isolated from intestinal bacteria; however, BSH activity of bacteria in bile-salt-free (non-intestinal) environments is largely unknown. Here, we aimed to identify BSH from non-intestinal *Enterococcus faecium* and characterize its enzymatic function. We successfully isolated a plasmid-encoded *bsh* (*efpBSH*) from *E. faecium*, and the recombinant EfpBSH showed BSH activity that preferentially hydrolyzed taurine-conjugated bile salts, unlike the activity of known BSHs. EfpBSH functioned optimally at pH 4.0 and 50 °C. EfpBSH exhibited very low amino acid sequence similarity (48.46%) to EfBSH from *E. faecalis* T2 isolated from human urine, although 241 sequences with 100% identity to EfpBSH were found in both plasmids and chromosomes of *E. faecium* strains inhabiting intestinal and non-intestinal environments. Phylogenetically, EfpBSH was not affiliated with any known BSH phylogroup and was clearly distinguished from previously identified BSHs from intestinal lactic acid bacteria. Our genome database analysis demonstrated that horizontal gene transfer causes global *efpBSH* distribution among *E. faecium* strains in various environments (soil, water, and intestinal samples) and geographical regions (Asia, Africa, Europe, North America, South America, and Australia/Oceania). Overall, our findings are the first to indicate that BSH is not an intestine-specific enzyme and that hitherto-overlooked probiotic candidates with BSH activity can exist in diverse environments.

## 1. Introduction

*Enterococcus faecium* is widely distributed across diverse environments including soil, river surface water, clinical settings, and mammalian intestines [1]. The physiological characteristics of the intestinal *E. faecium* vary depending on the strain. Some *E. faecium* strains are pathogenic, but others are non-pathogenic and exhibit probiotic potential, especially in terms of cholesterol-assimilating and anti-hypercholesterolemic capabilities [2,3,4]. Bile salt hydrolase (BSH; also known as choloylglycine hydrolase; EC3.5.1.24), produced by intestinal bacteria, is a key enzyme responsible for cholesterol-lowering and anti-hypercholesterolemic effects [5]. Among the intestinal bacteria, *E. faecium* is one of the species with the highest BSH activity [6].

The genes (*bsh*) encoding BSH are thought to be exchanged through horizontal gene transfer (HGT) among intestinal microbes, including probiotic bacteria (e.g., *Lactobacillus* spp. and *Bifidobacterium* spp.) and methanogenic archaea [7,8,9,10]. Furthermore, a well-characterized probiotic bacterium, *Ligilactobacillus salivarius* (formerly *Lactobacillus salivarius*) strain UCC118 [11], harbors two distinct *bsh* genes, one located on the chromosome and the other on a plasmid [12]. The plasmid-encoded *bsh* showed significantly high sequence similarities with both plasmid- [12] and chromosomal-encoded [13] *bsh* from intestinal *L. salivarius* strains (>93% similarity). While HGT of *bsh* is commonly observed in the intestine, little is known about whether HGT of *bsh* occurs in non-intestinal environments.

Although bile salts (BSH substrates) mainly exist in mammalian intestines, bacteria with BSH activity have recently been found in non-intestinal bacteria dwelling in the rhizosphere, hot water springs, Antarctic lakes, marine sediments, flowers, and fermented spider plants [14,15,16,17,18]. Due to lack of experimental data, the enzymatic function of BSH in these bacteria remains largely unclear. Notably, Singhal et al. reported that the BSH-producing *E. faecium* strain LR2, isolated from rhizospheric soil, exhibited high cholesterol-lowering capability and probiotic potential [14]. This finding suggests that non-intestinal *E. faecium* strains with BSH activity are promising probiotic candidates. To further support this hypothesis, biochemical characterization of BSH derived from non-intestinal bacteria and its diversity analysis are required.

In the present study, we aimed to explore a plasmid-encoded *bsh* from non-intestinal *E. faecium* based on genome database analysis. Notably, we succeeded in isolating a novel BSH (designated EfpBSH) with unique features, including rare substrate specificity and phylogenetic novelty, compared to previously identified BSHs from intestinal bacteria. Additionally, we investigated the geographical distribution of *efpBSH* among *E. faecium* strains using comparative genomic analyses and database searches.

## 2. Results and Discussion

### 2.1. Screening and Heterologous Expression of a Plasmid-Encoded bsh

**Figure 1:**
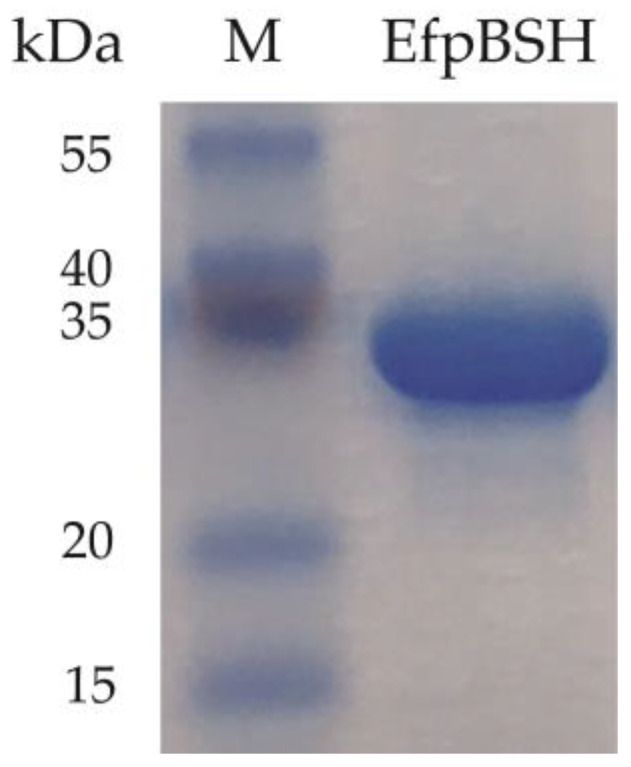
SDS-PAGE of purified EfpBSH. Lane M; 3-Color Prestained XL-Ladder (APRO Science, Tokushima, Japan).

Based on the sequence and domain search analyses, we identified three putative *bsh* in the plasmid sequences of *Enterococcus faecium* (strains F17E0263, N56454, and F39). These putative *bsh* shared both 100% nucleotide and amino acid sequence identities. We further identified that a putative plasmid-encoded *bsh* of *E. faecium* QU50 [19,20] exhibited 99.69% nucleotide sequence identity with the above three putative *bsh* sequences and shared 100% amino acid sequence identity with them. The difference in gene sequences was due to three silent mutations (_Leu_225_Leu_, _Gly_260_Gly_, and _Thr_262_Thr_). These four *E. faecium* strains were isolated from not only intestinal environments (young chickens and retail chickens in the USA [strain F17E0263 and N56454]) but also non-intestinal habitats (river surface water in Switzerland [strain F39] and eutrophic soil in Egypt [strain QU50]), suggesting that the plasmid-encoded *bsh* candidate (named *efpBSH*) can spread among *E. faecium* strains across continents. To investigate whether *efpBSH* acts as a functional BSH, we commercially synthesized *efpBSH*, constructed a heterologous *efpBSH* overexpression system, and purified the recombinant EfpBSH. The molecular weight of the purified His_6_-EfpBSH was approximately 35.0 kDa in size, which is almost the same as the theoretical molecular weight of EfpBSH consisting of 325 amino acids (Figure 1).

### 2.2. Enzymatic Activity of Recombinant EfpBSH

**Figure 2:**
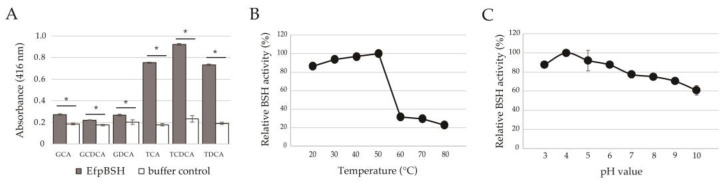
Enzymatic characterization of EfpBSH. (**A**) BSH activity and substrate specificity of EfpBSH. The tested substrates were glycocholic acid (GCA), glycochenodeoxycholic acid (GCDCA), glycodeoxycholic acid (GDCA), taurocholic acid (TCA), taurochenodeoxycholic acid (TCDCA), and taurodeoxycholic acid (TDCA). Each bile salt solution was mixed with buffer instead of EfpBSH (white bars) for specific negative controls. Values are indicated as means of five technical replicates (*n* = 5). Error bars represent standard deviation (SD). Identification of the effects of temperature (**B**) and pH (**C**) on BSH activity. The tested temperature and pH ranges were 20–80 °C and pH 3.0–10.0, respectively. Each value is expressed as the mean of three technical replicates (*n* = 3). Maximum activity was taken as 100%. Error bars indicate SD. For all analyses, a *p*-value less than 0.05 (* *p* < 0.05) was defined as statistically significant.

The BSH activity of the purified EfpBSH on six major human bile salts, including three glycine-conjugated (glycocholic acid [GCA], glycochenodeoxycholic acid [GCDCA], and glycodeoxycholic acid [GDCA]) and three taurine-conjugated (taurocholic acid [TCA], taurochenodeoxycholic acid [TCDCA], and taurodeoxycholic acid [TDCA]) isomers, was measured. EfpBSH hydrolyzed all the tested conjugated bile salts. In particular, BSH activity on taurine-conjugated bile salts was much higher than that on glycine-conjugated bile salts (Figure 2A), indicating that EfpBSH is a functional BSH with high specificity for taurine-conjugated bile salts. Notably, BSHs from probiotic lactic acid bacteria generally tend to deconjugate glycine-conjugated bile salts rather than taurine-conjugated bile salts [21], suggesting that EfpBSH has uncommon substrate specificity. To our knowledge, this is the first report on the isolation and enzymatic characterization of plasmid-encoded *bsh* in *E. faecium*.

We determined the effects of temperature and pH on the enzymatic activity of EfpBSH. The optimum temperature and pH of EfpBSH were 50 °C (Figure 2B) and pH 4.0 (Figure 2C), respectively. EfpBSH exhibited stable activity and maintained approximately >80% of its original activity at mesophilic conditions (20–50 °C) and mildly acidic conditions (pH 3.0–6.0), whereas lower BSH activities were exhibited at high temperature ranges (60–80 °C) and mild alkaline (pH 8.0–10.0) conditions (Figure 2B,C). We found that EfpBSH has pH preference similar to most known BSHs from lactic acid bacteria, which generally function optimally at a mildly acidic pH range (pH 3.8–5.0) [22]. EfpBSH is relatively thermostable compared to previously identified BSHs, which act optimally at approximately 37 °C [22]. Thus, we identified the enzymatic activity and biochemical characteristics of the recombinant EfpBSH.

### 2.3. Sequence and Phylogenetic Analyses of EfpBSH

**Figure 3:**
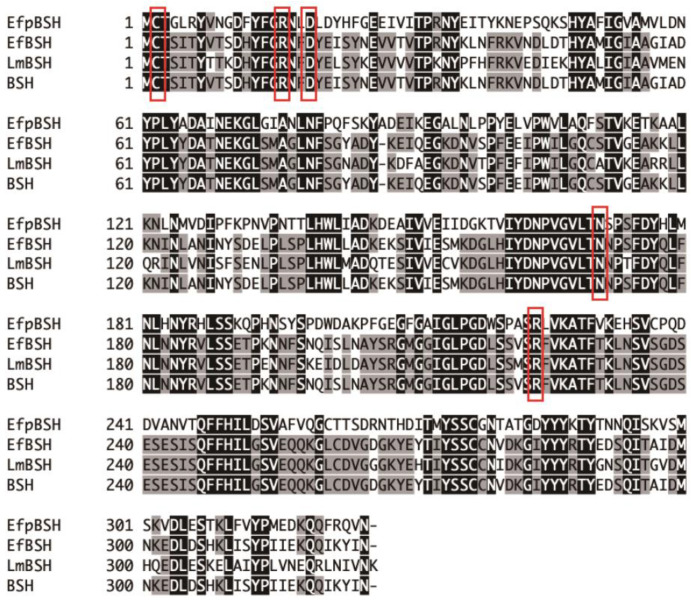
Multiple alignment analysis of EfpBSH. The amino acid sequence of EfpBSH was compared with BSHs from *Enterococcus faecalis*, *Listeria monocytogenes*, and *Enterococcus faecium*. The black and gray backgrounds indicate identical and similar amino acid residues, respectively. The conserved amino acid residues (Cys, Arg, Asp, Asn, and Arg) associated with the catalytic active site are boxed in red lines. Sources of BSHs: EfBSH (4WL3) from *E. faecalis* T2 [23]; LmBSH (QHF62338) from *L. monocytogenes* EGD-e [24]; and BSH from *E. faecium* LR2 [14].

Amino acid sequence comparison of EfpBSH with previously identified enzymes revealed that EfpBSH has the highest but still very low sequence similarity (48.46%) to EfBSH from *E. faecalis* T2 [23], followed by LmBSH from *Listeria monocytogenes* EGD-e [24] (48.0%) and BSH from *E. faecium* LR2 (47.69%) [14] (Table 1). In contrast, the chromosomal BSHs from *Enterococcus* bacteria (T2 and LR2) and *L. monocytogenes* shared much higher amino acid sequence similarity (73.68–82.04%). We found that five amino acid residues (Cys-2, Arg-16, Asp-19, Asn-171, and Arg-224 in EfpBSH) associated with the active site and the catalytic reaction were completely conserved (Figure 3). These results suggest that EfpBSH is a novel BSH with low sequence similarity to previously identified BSHs.

**Figure 4:**
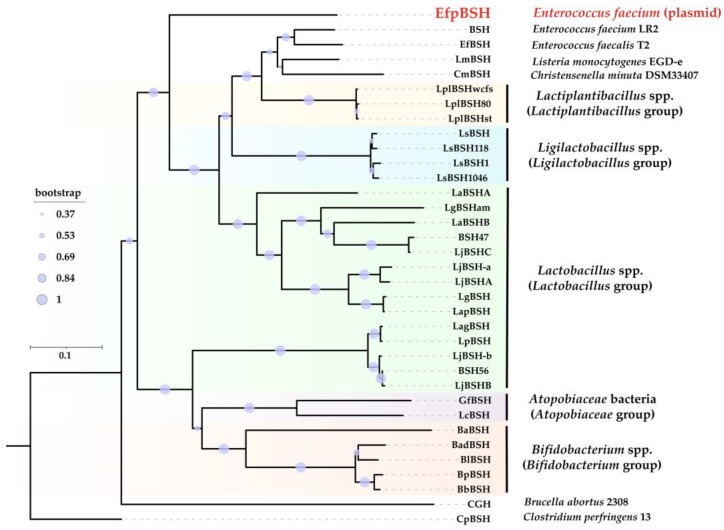
Phylogenetic analysis of EfpBSH. The phylogenetic tree was constructed using MEGA X software version 10.1.8 based on the neighbor-joining method (1000 bootstrap replications). Bootstrap values are represented by circles, whose sizes correlate with the bootstrap values. Each enzyme name was defined based on the names of genus, species, and strain (Table 1). EfpBSH was indicated in red. Each BSH group was highlighted by different background color. CpBSH, BSH from *Clostridium perfringens* 13, was used as the outgroup.

Phylogenetic analysis results further highlighted the novelty of EfpBSH. As shown in Figure 4 and Figure S1, the phylogenetic position of each BSH was generally grouped based on its BSH-retaining bacterial taxa, including *Atopobiaceae*, *Bifidobacterium*, *Lactiplantibacillus*, *Lactobacillus*, and *Ligilactobacillus* groups. Notably, EfpBSH was not affiliated with any known BSH phylogroup and was deeply branched from all previously characterized BSHs, supported by high bootstrap values. This indicates that EfpBSH is a phylogenetically novel BSH.

Furthermore, we found 241 amino acid sequences with 100% identity to EfpBSH (4 plasmid-encoded and 237 chromosome-encoded sequences) in *E. faecium* genomes based on the NCBI database search (see Supplementary Table S1). These 241 EfpBSH sequences were derived not only from microbes inhabiting intestine-related environments but also from those inhabiting other natural environments, including soil and water. More importantly, these EfpBSH sequences were found in various countries and regions, including the Asian continent (Bangladesh, China, Israel, and Pakistan), Africa (Egypt, Ghana, and South Africa), Europe (Belgium, Denmark, Estonia, France, Germany, Netherlands, Portugal, Russia, Switzerland, and Sweden), North America (Canada, Saint-Pierre, and USA), South America (Brazil), and Australia/Oceania (Australia and New Zealand) (Supplementary Table S1). The results suggest that the *efpBSH*-related genes are widely conserved in *E. faecium* genomes (both plasmids and chromosomes) through HGT.

**Figure 5:**
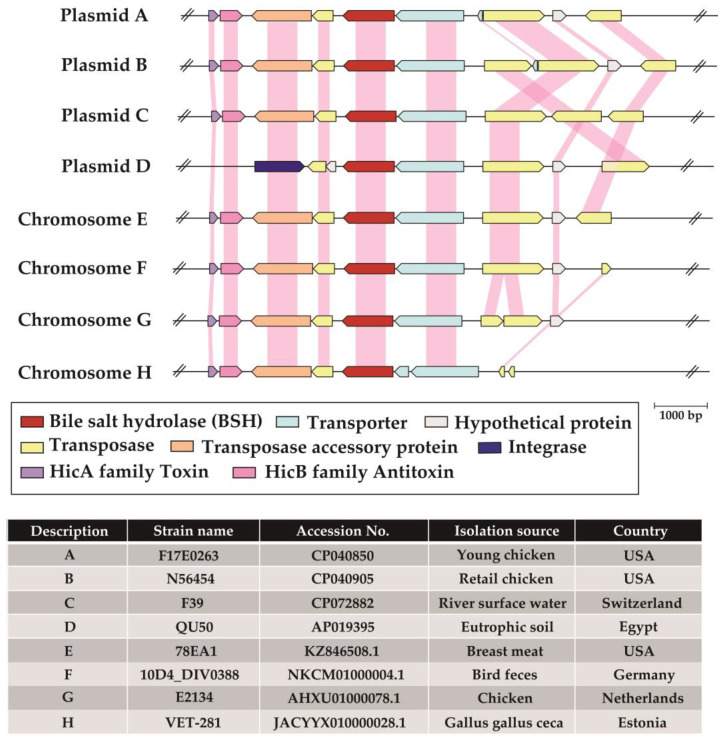
Comparative genomic analysis of *efpBSH*-encoding plasmids and chromosomes in *E. faecium* strains. A physical map and gene organization of *efpBSH* and their surrounding genes are provided (reversed organization in plasmid B and chromosome E and F). Each sequence’s information is listed in the table. Light pink shadings indicate homologous genes with >95% amino acid sequence similarity. The ORFs are color-coded according to predicted protein functions provided in the middle panel. The scale bar indicates 1000 bp.

We further performed comparative genomic analysis of four *efpBSH*-encoding plasmids and four selected chromosomes in *E. faecium* strains. Although the genes were derived from completely different isolation sources and countries, *efpBSH* and its surrounding genes were highly conserved (Figure 5). In particular, conjugated bile salt major facilitator superfamily transporter genes, which play an important role in the uptake of conjugated bile salts, were noted in all sequences and were located downstream of *efpBSH*. Elkins et al. reported that the genes encoding BSH and bile salt transporters probably constitute operons [8,25]. Furthermore, we observed that the type II toxin–antitoxin gene operon was highly conserved in these sequences. Type II toxin–antitoxin systems have been widely found in plasmids and function as addiction modules (plasmid maintenance) through a phenomenon called post-segregational killing of plasmid-free cells [26]. These systems likely play a critical role in maintaining *efpBSH*-encoding plasmids in *E. faecium* strains. Additionally, an integrase-related gene, several transposon-related genes (ISEfm1, ISEfa4, IS256, IS1216, IS30, and IS6 family transposases), and transposon-accessory genes were noted near the *efpBSH* genes (Figure 5). Mobile genetic elements (e.g., plasmids, integrative and conjugative elements, and transposons) exert an important role in HGT among bacteria and facilitate adaptation to the environments [27]. Notably, not only BSH but also other genes related to plasmid maintenance and mobile genetic elements shared high (>95%) similarity among *E. faecium* strains (Figure 5). Therefore, these gene regions, including *efpBSH* and its surrounding genes, could be exchanged through HGT among both intestinal and non-intestinal *E. faecium*.

The reason for the maintenance of *efpBSH* among some non-intestinal *E. faecium* strains remains unclear. BSHs are structurally and phylogenetically similar to penicillin V acylase (EC3.5.1.11) [28,29] and acyl-homoserine lactone acylase (AHL-acylase, EC3.5.1.97) [30]. Notably, a few BSHs from *Lactiplantibacillus plantarum* degrade AHLs (cell–cell communication signals in quorum-sensing systems) [31]. AHLs regulate various physiological functions in bacteria, such as virulence [32], biofilm formation [33], and antibiotic production [34]; therefore, in competitive bacteria with AHL-acylase, degradation of AHL and quenching of the quorum-sensing system by AHL-acylase would confer ecological advantages of enhanced survival and better colonization in the environment. Furthermore, Kusada et al. recently reported the novel function of BSH enzymes (LpBSH and LapBSH) from a probiotic bacterium *Lactobacillus paragasseri* JCM 5343^T^, and demonstrated their bi-functional activities capable of degrading not only bile salts but also the antibiotic (penicillin) via penicillin V acylase activity [28,29]. Therefore, EfpBSH may confer antibiotic resistance to non-intestinal *E. faecium* strains, thus facilitating their survival in natural environments. However, further studies are required to verify this hypothesis.

## 3. Materials and Methods

### 3.1. bsh Synthesis, Cloning, and Heterologous Expression

All experiments were performed according to our previous studies [28,29,35,36]. In brief, we commercially synthesized a *bsh* (*efpBSH*) sequence (GenScript, Piscataway, NJ, USA). The OptimumGene^TM^ algorithm software (GenScript) was used to optimize various parameters, including codon usage bias and GC content, which are critical for efficient heterologous gene expression in *Escherichia coli* (https://www.genscript.com/gensmart-free-gene-codon-optimization.html) (accessed on 6 January 2022). *efpBSH* was cloned into the NdeI and EcoRI sites of the pCold II vector (ampicillin resistant, TaKaRa, Tokyo, Japan) and transformed into *E. coli* BL21 (DE3) competent cells (SMOBIO Technology Inc., Hsinchu, Taiwan). When the OD_600_ value reached 0.5–0.6, the culture medium was statically incubated at 15 °C for 30 min, and then isopropyl-β-D-thiogalactopyranoside (Nacalai Tesque Inc., Kyoto, Japan) was added to the culture medium at the final concentration of 100 μM. Subsequently, *E. coli* cells were cultured overnight at 15 °C with shaking. The cell pellet was collected by centrifugation at 5800× *g* for 10 min using a fixed-angle rotor, suspended in lysis buffer (20 mM Tris-HCl, 150 mM NaCl, 10% glycerin, and 5 mM imidazole; pH 7.0), and disrupted for 5 min via sonication using an ultrasonic disintegrator (BRANSON Sonifier 250; Branson, Danbury, CT, USA) in an ice-water bath. The soluble-fraction-containing His_6_-tagged protein was further purified via affinity open-column chromatography using Ni-NTA agarose HP (FUJIFILM Wako Pure Chemical Corporation, Tokyo, Japan). The purified fraction was dialyzed in a buffer using a semipermeable membrane (Spectra/Por 3 membrane MWCO: 3500; Repligen, Waltham, MA, USA). Standard sodium dodecyl sulfate–polyacrylamide gel electrophoresis (SDS-PAGE) was performed using a Mini-PROTEAN TGX precast polyacrylamide gel (Bio-Rad, Hercules, CA, USA).

### 3.2. BSH Activity

The BSH assay was performed using a standard colorimetric determination method as described previously [28,29,36,37,38]. The tested substrates were glycocholic acid (GCA, Cayman Chemical, Ann Arbor, MI, USA), glycochenodeoxycholic acid (GCDCA; Sigma-Aldrich, St. Louis, MO, USA), glycodeoxycholic acid (GDCA; Sigma-Aldrich), taurocholic acid (TCA; Nacalai Tesque), taurochenodeoxycholic acid (TCDCA; Sigma-Aldrich), and taurodeoxycholic acid (TDCA, Nacalai Tesque). Statistical analysis (Student’s *t*-test) was performed using GraphPad Prism software (version 8.0; GraphPad Software, San Diego, CA, USA). Five technical experiments were performed (*n* = 5). For all analyses, a *p*-value less than 0.05 (*p* < 0.05) was defined as statistically significant.

### 3.3. Determination of Optimum Enzymatic Condition

All experiments were performed according to our previous studies [28,29,36]. We selected TDCA as a representative substrate to determine the optimum enzymatic conditions for EfpBSH. The tested temperatures and pH ranges were 20–80 °C (in intervals of 10 °C) and pH 3.0–10.0 (at intervals of pH 1.0), respectively. The following Good’s buffer solutions were used to determine the effects of pH on enzyme activity: acetate (pH 3.0–4.0, FUJIFILM Wako Pure Chemical Corporation); MES (pH 5.0–6.0, Dojindo Laboratories, Kumamoto, Japan); HEPES (pH 7.0–8.0, Dojindo Laboratories); and CAPS buffers (pH 9.0–10.0, Dojindo Laboratories). All experiments were performed in triplicate (*n* = 3).

### 3.4. Phylogenetic Analysis

Amino acid sequences of the experimentally identified BSHs were used in this analysis (accession numbers are provided in Table 1). A phylogenetic tree was constructed using MEGA X (https://www.megasoftware.net/) based on the neighbor-joining and maximum-likelihood methods, with 1000 bootstrap replicates [39]. The phylogenetic tree was further analyzed and visualized using the online tool, Interactive Tree Of Life (iTOL v6; https://itol.embl.de/) [40]. The evolutionary distances were computed using the Poisson correction method, and they were presented as the number of amino acid substitutions per site [41] (accessed on 15 August 2023).

### 3.5. Sequence and Database Searches

Multiple alignment analysis of EfpBSH was performed using GENETYX-MAC software version 21.2.0 (GENETYX Corp., Tokyo, Japan) and the amino acid sequences of previously identified BSHs from *Enterococcus faecalis*, *Listeria monocytogenes*, and *E. faecium*. Four plasmid-encoded *efpBSH* sequences (accession numbers CP040850, CP040905, CP072882, and AP019395) were obtained from the National Center for Biotechnology Information database (https://www.ncbi.nlm.nih.gov/). Information about the geographic location, host bacterium, and isolation source of proteins identical to EfpBSH were acquired from the protein (https://www.ncbi.nlm.nih.gov/protein/, accessed on 15 August 2023) and biosample (https://www.ncbi.nlm.nih.gov/biosample/, accessed on 15 August 2023) databases. Comparative genomic analysis was conducted using four *efpBSH*-encoding plasmids and four selected chromosomes in *E. faecium* strains and visualized using the online tool, drawGeneArrows3 (http://www.ige.tohoku.ac.jp/joho/index.html) (accessed on 15 August 2023).

## 4. Conclusions

In the present study, we successfully isolated a novel BSH (EfpBSH) from a plasmid of *E. faecium* and clarified its unique enzymatic and phylogenetic features. Unlike known BSH enzymes, the recombinant EfpBSH exhibited significantly higher activity on taurine-conjugated bile salts (TCA, TCDCA, and TDCA) compared to glycine-conjugated bile salts (GCA, GCDCA, and GDCA), indicating that EfpBSH possesses rare substrate specificity. In addition, we found that EfpBSH is a phylogenetically novel BSH with a low amino acid sequence similarity to previously identified BSHs. Our analysis further indicates the geographical distribution of *efpBSH* through HGT among *E. faecium* strains.

Overall, this is the first study to demonstrate the enzymatic function of BSH derived from non-intestinal *E. faecium* (and its plasmid), and to indicate the potential worldwide distribution of *bsh* in *E. faecium* through HGT. Our findings suggest that previously overlooked probiotic bacteria with *bsh* (functionally rare and/or phylogenetically novel) may exist in non-intestinal environments, making these environments new targets for screening yet-to-be-isolated *bsh* genes. Our study could serve as a basis for further research on the ecological advantages of environmental bacteria with BSH and screening probiotic bacteria with BSH in non-intestinal environments.

## Figures and Tables

**Table 1:** Amino acid sequences of identified BSHs.

Enzyme	Accession Number	Strain	Similarity to EfpBSH (%)
EfBSH	4WL3	*Enterococcus faecalis* T2	48.46
LmBSH	QHF62338	*Listeria monocytogenes* EGD-e	48.00
BSH	- *	*Enterococcus faecium* LR2	47.69
CmBSH	QXL43068	*Christensenella minuta* DSM33407	44.62
CpBSH	P54965	*Clostridium perfringens* 13	42.86
LapBSH	BBD48841	*Lactobacillus paragasseri* JCM 5343	47.35
LpBSH	BBD4770		40.44
LgBSH	WP_020806888	*Lactobacillus gasseri* FR4	46.79
LjBSHA	EGP12224	*Lactobacillus johnsonii* PF01	45.26
LjBSHB	ABQ01980		40.75
LjBSHC	EGP12391		43.03
LjBSH-a	AAG22541	*Lactobacillus johnsonii* 100-100	45.26
LjBSH-b	AAC34381		41.07
LaBSHA	AAV42751	*Lactobacillus acidophilus* NCFM	43.25
LaBSHB	AAV42923		43.87
BSH47	Q74JG0_LACJO	*Lactobacillus johnsonii* CNCM I-12250	43.03
BSH56	Q74LX7_LACJO		41.07
LgBSHam	ACL98172	*Lactobacillus gasseri* AM1	42.33
LagBSH	ABJ59469	*Lactobacillus gasseri* JCM 1131	40.75
LsBSH1	ACL98197	*Ligilactobacillus salivarius* LGM14476	44.31
LsBSH1046	ACL98194	*Ligilactobacillus salivarius* JCM 1046	43.38
LsBSH	AFP87505	*Ligilactobacillus salivarius* NRRL B-30514	44.00
LsBSH118	ACL98201	*Ligilactobacillus salivarius* UCC118	41.85
LplBSHst	ADO00098	*Lactiplantibacillus plantarum* subsp. *plantarum* ST-III	44.92
LplBSH80	AAB24746	*Lactiplantibacillus plantarum* 80	44.62
LplBSHwcfs	CAD65617	*Lactiplantibacillus plantarum* WCFS1	44.92
BbBSH	AAR39435	*Bifidobacterium bifidum* ATCC 11863	37.90
BlBSH	AAF67801	*Bifidobacterium longum* SBT2928	36.62
BadBSH	AAX86039	*Bifidobacterium adolescentis* ATCC 15705	36.08
BpBSH	KFI75916	*Bifidobacterium pseudocatenulatum* DSM 20438	37.26
BaBSH	AAS98803	*Bifidobacterium animalis* subsp. *lactis* KL612	33.65
LcBSH	BDC90728	*Leptogranulimonas caecicola* TOC12	33.56
GfBSH	GJM54962	*Granulimonas faecalis* OPF53	32.79
CGH	CAJ11444	*Brucella abortus* 2308	32.52

* The sequence is not registered in the gene bank database of NCBI, but the whole amino acid sequence of BSH is provided in the text of the paper [14].

## Data Availability

Data is contained within the article and Supplementary Materials.

## Supplementary Materials

The following supporting information can be downloaded at: https://www.mdpi.com/article/10.3390/ijms26020612/s1.
