# Supplementary material for: Evidence for the Worldwide Distribution of a Bile Salt Hydrolase Gene in Enterococcus faecium Through Horizontal Gene Transfer
Source: Int J Mol Sci. 2025 Jan 13;26(2):612. doi: 10.3390/ijms26020612 (PMC11765501; doi:10.3390/ijms26020612)
Supplement: Supplementary file 1 [file ijms-26-00612-s001.zip › Figure S1 Kusada et al.pdf]

Evidence for the Worldwide Distribution of a Bile Salt Hydrolase Gene in *Enterococcus faecium* Through Horizontal Gene Transfer

Hiroyuki Kusada * and Hideyuki Tamaki *

Bioproduction Research Institute, National Institute of Advanced Industrial Science and Technology, Tsukuba 305-8566, Ibaraki, Japan

* Correspondence: kusada-hiroyuki@aist.go.jp (H.K.); tamaki-hideyuki@aist.go.jp (H.T.); Tel.: +81-29-861-6591 (H.K. & H.T.); Fax: +81-29-861-6587 (H.K. & H.T.)

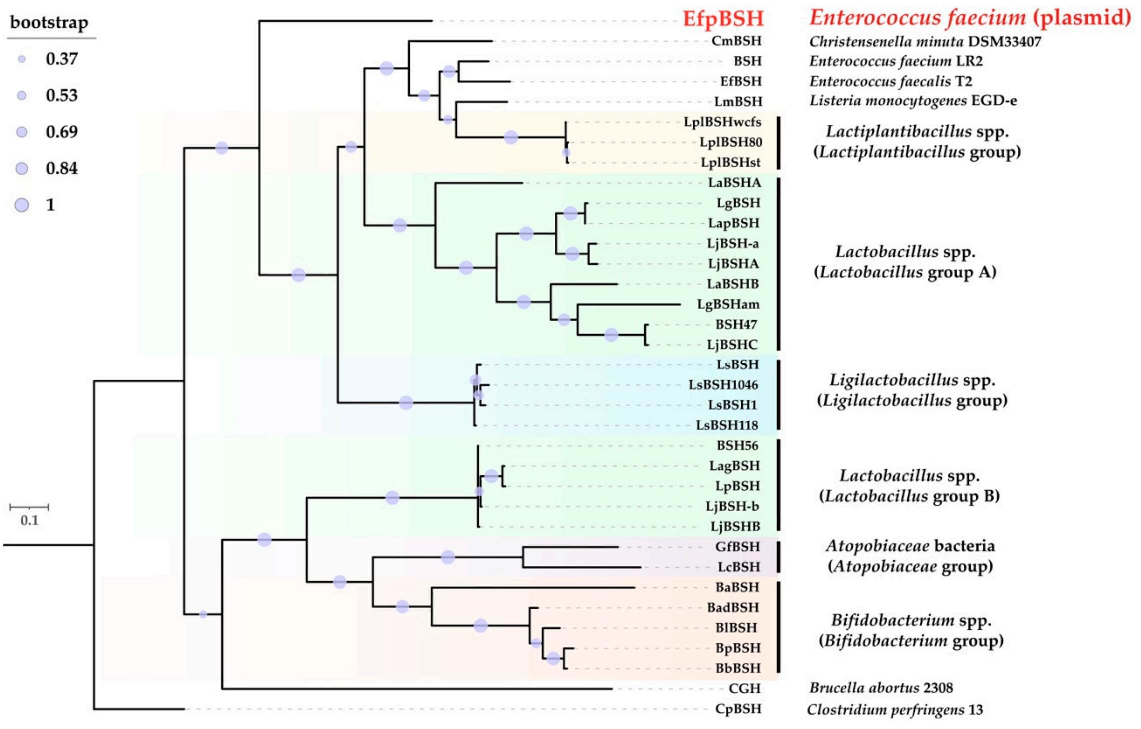

Figure S1. Phylogenetic analysis of EfpBSH. The phylogenetic tree was constructed using MEGA X software version 10.1.8 based on the maximum-likelihood method (1000 bootstrap replications). Bootstrap values are represented by circles, whose sizes correlate with the bootstrap values. Each enzyme name was defined based on the names of genus, species, and strain (Table 1). EfpBSH was indicated in red. Each BSH group was highlighted by different background color. CpBSH, BSH from *Clostridium perfringens* 13 was used as the outgroup.
